# Device-measured sedentary time in Norwegian children and adolescents in the era of ubiquitous internet access: secular changes between 2005, 2011 and 2018

**DOI:** 10.1093/ije/dyac063

**Published:** 2022-04-01

**Authors:** Knut Eirik Dalene, Elin Kolle, Jostein Steene-Johannessen, Bjørge H Hansen, Ulf Ekelund, May Grydeland, Sigmund Alfred Anderssen, Jakob Tarp

**Affiliations:** Department of Sports Medicine, Norwegian School of Sports Sciences, Oslo, Norway; Department of Sports Medicine, Norwegian School of Sports Sciences, Oslo, Norway; Department of Sports Medicine, Norwegian School of Sports Sciences, Oslo, Norway; Department of Sports Medicine, Norwegian School of Sports Sciences, Oslo, Norway; Department of Sport Science and Physical Education, University of Agder, Kristiansand, Norway; Department of Sports Medicine, Norwegian School of Sports Sciences, Oslo, Norway; Department of Physical Performance, Norwegian School of Sports Sciences, Oslo, Norway; Department of Sports Medicine, Norwegian School of Sports Sciences, Oslo, Norway; Department of Sports Medicine, Norwegian School of Sports Sciences, Oslo, Norway

**Keywords:** Sedentary, physical activity, screen-time, children, adolescents, accelerometer, secular change

## Abstract

**Background:**

Access to screen-based media has been revolutionized during the past two decades. How this has affected sedentary time (ST) accumulation in children is poorly understood.

**Methods:**

This study, based on the Physical Activity among Norwegian Children Study (PANCS), uses accelerometer data from population-based samples of 9- and 15‐year-olds, collected in 2005 (*n* = 1722), 2011 (*n* = 1587) and 2018 (*n* = 1859). Secular changes between surveys were analysed using random-effects linear regression models adjusted for survey-specific factors. Data on ST were collected using hip-worn ActiGraphs and ST was defined using a threshold equivalent to <100 counts/min. Sedentary bouts were grouped by duration: <1, 1–5, 5–15, 15–30 and ≥30 min.

**Results:**

Between 2005 and 2018, ST increased by 29 min/day in 9-year-old boys (95% CI: 19, 39; *P *<0.001), by 21 min/day in 15-year-old boys (95% CI: 8, 34; *P *=* *0.002) and by 22 min/day in 15-year-old girls (95% CI: 10, 35; *P *<0.001), but not in 9-year-old girls at 6 min/day (95% CI: -3, 16; *P *=* *0.191). All age-sex groups accumulated less ST in bouts lasting <5 min and more ST in longer bouts, particularly in 5–15-min bouts. Adolescent girls also increased ST accumulation in 15–30-min and ≥30-min bouts. Changes were largely mirrored before, during and after school on weekdays and during weekend days.

**Conclusions:**

Coinciding with the introduction of smartphones, tablets and near-universal internet access, total daily ST and ST accumulated in prolonged sedentary bouts increased between 2005 and 2018 in children and adolescents.

Key Messages

- Sedentary time is a risk factor for morbidity and early mortality, with prolonged bouts of sedentary time suggested as particularly detrimental.
- Technological advancements have revolutionized access to screen-based media during the past two decades, but how this has affected the total volume and accumulation pattern of sedentary time in children and adolescents is poorly understood.
- We used accelerometer data from population-based samples of 9- and 15‐year-olds collected in 2005, 2011 and 2018, to examine secular changes in sedentary time from before to during and after the introduction of smartphones, tablets and near-universal internet access.
- We found that sedentary time increased by 20-30 min/day in 9-year-old boys and in 15-year-olds from 2005 to 2018, and that all age-sex groups spent less time in sedentary bouts lasting <5 min and more time in longer bouts of uninterrupted sedentary time.
- Our data suggest that contemporary children and adolescents are spending a larger part of their day in uninterrupted sedentary pursuits than before the introduction of smartphones, tablets and near-universal internet access.

## Introduction

A high level of sedentary time (ST) is a risk factor for morbidity and early mortality.^1^ The newly updated physical activity and sedentary behaviour guidelines from the World Health Organization (WHO) now suggest that individuals should limit ST across the life course.^1^ Whereas a sufficient level of physical activity may counteract the detrimental effects of high volumes of daily ST,^2^ many children and adolescents do not meet the recommended minimum of physical activity.^3^ The adverse consequences of ST may not only be determined by the total volume, but also by its accumulation pattern, with prolonged bouts of ST suggested as particularly detrimental.^4^

The advent of available and affordable technology and digital communication during the past 20 years is considered to have fundamentally changed the way children and adolescents engage in screen-based behaviours.^5–8^ In the USA, 95% of teenagers had access to a smartphone in 2018, an increase from 73% just 3 years earlier.^7^ Similar trends are mirrored in other Western countries, including Norway.^6^ Yet, it remains uncertain how this has impacted on both the total volume and the pattern of ST accumulation, with only one previous study having assessed secular changes in (total) ST, using activity monitors.^9^ Device-based data are a prerequisite for monitoring secular trends in ST accurately,^10^ as it is possible that new screen-based behaviours have simply replaced other sedentary pursuits.^8^ Accordingly, epidemiological studies describing secular trends in both the total volume of ST and how ST is accumulated through different bout durations are needed to monitor population exposure and guide public health action.

The Physical Activity among Norwegian Children Study (PANCS) is a serial, cross-sectional survey of accelerometer-assessed physical activity and ST, designed to monitor secular changes between nationally representative samples of children and adolescents, and serves as the national physical activity and ST surveillance system in Norway. Because the first survey was initiated in 2005, we are in a unique position to perform a detailed comparison of ST before (2005), during (2011) and after (2018) the introduction of smartphones, tablets and near-universal internet access. We therefore aim to describe secular changes in total and patterned ST in 9- and 15-year-old children and adolescents. As a secondary aim, we explore if changes are similar on weekdays and weekend days and during time-segments on schooldays (before, during, after school hours).

## Methods

Three waves of the Physical Activity among Norwegian Children Study have been conducted by the Norwegian School of Sport Sciences in collaboration with the Norwegian Directorate for Health and the Norwegian Institute of Public Health, in 2005 (PANCS1), 2011 (PANCS2) and 2018 (PANCS3). All study waves were carried out in accordance with the Declaration of Helsinki. The Regional Committee for Medical Research Ethics (RCMRE) approved PANCS1, whereas PANCS2 and PANCS3 were considered outside the Health Research Act's scope by the RCMRE and therefore not considered subject to approval. The Norwegian Centre for Research Data^11^ approved the processing of personal data in all three study waves. Participants and their parents/legal guardians signed written informed consent forms before data collections. The methods used in the PANCS data collections are described in detail elsewhere,^12^ and briefly summarised below.

### Study sample

In 2005 (PANCS1), Statistics Norway selected nationally representative samples of 9- and 15-year-olds (4th and 10th graders) using cluster sampling with schools as the primary unit, taking geography and population density into account. In 2011 (PANCS2), a mixed design was used. Statistics Norway selected a new, nationally representative sample of 9-year-olds using the same method as in PANCS1. Further, 15-year-olds were either invited individually, based on previous participation in PANCS1 at age 9 years (*n *= 1119), or from a random sample of schools that had previously participated in PANCS1 (*n *= 640). In 2018 (PANCS3), we invited the same schools that Statistics Norway had selected for PANCS1 (10th graders) and PANCS2 (4th graders). If a school declined to participate, we invited another school from the same, or a corresponding, geographical and sociodemographic area. When schools agreed to participate, we invited all 4th and/or 10th grade pupils to participate. The present study compares cross-sectional data from 9- and 15-year-olds participating in PANCS1, 2 or 3.

### Outcome assessment

We assessed physical activity and ST using hip-worn ActiGraph accelerometers (ActiGraph, Pensacola, FL, USA). PANCS1 used model CSA7164, PANCS2 used models GT1M and GT3X+, and models GT3X+ and GT3X+BT were used in PANCS3. Participants were instructed to wear the monitor during all waking hours for a full week in PANCS2 and 3, and for 4 consecutive days (including 2 weekend days) in PANCS1, due to limited monitor storage capacity of the CSA7164. All monitors were initialized to start recording at 06:00 the day after the participants received them.

The piezoelectric accelerometer within ActiGraph model CSA7164 records less ST than the Micro-electromechanical system-based accelerometers within the newer generations of ActiGraphs under default settings.^13^ Therefore, we reanalysed all files from PANCS2 and 3 with the low-frequency extension (LFE) filter activated. Enabling the LFE filter almost completely attenuates the inter-generation difference in ST recordings in both mechanical and free-living environments.^14^ We ascertained inter-generation comparability following LFE filtering by reanalysing data collected in 14 Norwegian 10-year-olds who wore CSA7164 and GT3X+ devices simultaneously over 3 full days (Supplementary Material, available as Supplementary data at *IJE* online, page 2).^13^ The PANCS2 participants who had ST assessed by the GT1M model (317 and 231 9- and 15-year-olds, respectively) were excluded from the analytical sample, as reanalysis of raw data with LFE filtering is not possible.

After excluding all data recorded between 00:00 to 06:00 and non-wear (defined as consecutive spells of zero counts lasting ≥60 min),^15^ we defined a valid day as ≥480 min of data,^16^ and included all participants with ≥1 weekday and ≥1 weekend day of valid activity recordings.^17^ In Norway, school normally starts between 8:00 and 9:00 and ends between 13:00 and 14:45, depending on school and grade. We did not have exact school start and end times, but summarized ST and sedentary patterns during the following segments on school-days: ‘morning’ (06:00 to 09:00), ‘school’ (09:00 to 13:00/09:00 to 14:00), and ‘after school’ (13:00 to 22:00/14:00 to 23:00) for 9- and 15-year-olds, respectively. Time recorded in these segments were deemed valid if they meet the inclusion criteria presented in the Supplementary Material (page 3).

We classified all 10-s epochs with <17 activity counts as ST, which is equivalent to the widely used ≤100 CPM cut-point.^18^ Secular changes in sedentary patterns were explored by deriving ST accumulated in uninterrupted sedentary bouts of 0 to 1, 1 to 5, 5 to 15, 15 to 30 and >30 min duration. Any epoch count value ≥17 resulted in termination of the bout.^19^

### Covariates

We considered age, seasonality (minutes of daylight on accelerometery start date), mean accelerometer wear time (min/day or min/segment) and weekday:weekend ratio (number of valid weekdays divided by number of valid weekend days) as relevant covariates.

### Statistical analyses

Secular trends in total ST and bouts of ST across the 2005, 2011 and 2018 surveys were analysed using random-effects linear regression, with school as the random effect and 95% confidence intervals adjusted for the school-level (cluster) sampling. All secular trend analyses were adjusted for age, minutes of daylight at the time of ST assessment and segment-specific accelerometer wear time as fixed effects, because between-survey differences in these variables may confound estimates of ST.^15^^,^^20^^,^^21^ For the same reason, all analyses combining data collected on weekdays and weekend days were additionally adjusted for the ratio of week- to weekend days.^22^ Secular trends in bouts of ST were not adjusted for concurrent trends in total ST. Thus, these data reflect a combination of changes in the composition of ST as well as changes in total ST. Model assumptions were verified by inspection of the distribution of predicted residuals. Statistical analyses and processing of the time-stamped accelerometer files were performed using Stata SE 16.0 (StataCorp, College Station, TX, USA).

## Results

**Figure 1:**
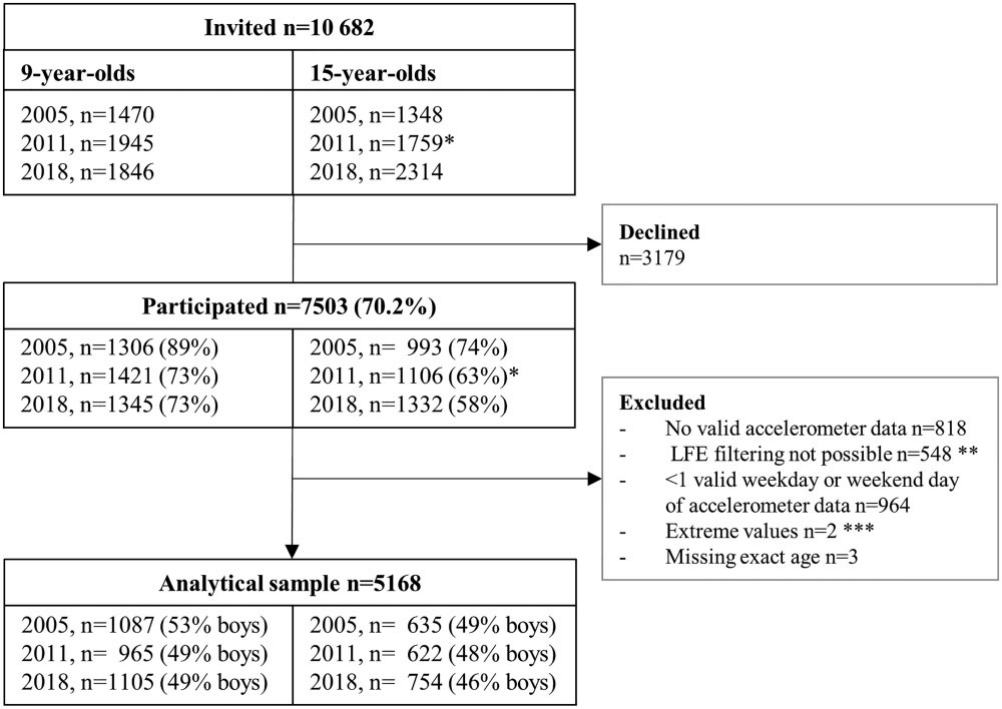
Numbers of 9- and 15-year-olds who were invited, participated and were included in the analytical sample of the present study by Physical Activity among Norwegian Children Study cohort and age group. *1119 of the 1306 that participated in Physical Activity among Norwegian Children Study 1 as 9-year-olds were located and invited to participate in Physical Activity among Norwegian Children Study 2 as 15-year-olds. Of these, 731 (65.3%) chose to participate. The remaining 640 15-year-olds who were invited to participate in Physical Activity among Norwegian Children Study 2 were from seven randomly selected lower secondary schools that had previously participated in Physical Activity among Norwegian Children Study 1. Of these, 375 (58.6%) chose to participate, yielding a combined total sample of 1106 15-year-olds in Physical Activity among Norwegian Children Study 2. **Applies only to Physical Activity among Norwegian Children Study 2 participants who wore a GT1M device (317 and 231 9- and 15-year-olds, respectively). ***Counts per minute values >2500. LFE, low frequency extension

Among 5261 and 5421 invited 9- and 15-year-olds overall, 4072 (77%) and 3331 (61%) agreed to participate, respectively (Figure 1). After excluding those not fulfilling the inclusion criteria (including the 548 PANCS2 participants who wore a GT1M device), we were able to harmonize and include data from 3157 9-year-olds and 2011 15-year-olds in our analytical samples. Table 1 displays background characteristics of those included by study year, age group and sex.

**Table 1:** Background characteristics and accelerometer wear compliance by study year, age group and sex

	Year	9-year-olds		15-year-olds	
		Boys	Girls	Boys	Girls
**Background characteristics**					
*n* ^a^	2005	579 (574)	508 (503)	308 (302)	327 (323)
	2011	474 (459)	491 (481)	296 (266)	326 (282)
	2018	541 (534)	564 (548)	344 (329)	410 (397)
Age (years)	2005	9.6 (0.38)	9.6 (0.39)	15.6 (0.35)	15.6 (0.36)
	2011	9.6 (0.44)	9.6 (0.43)	15.3 (0.64)^b^	15.2 (0.62)^b^
	2018	9.5 (0.37)^d^	9.5 (0.37)^d^	15.4 (0.37)^d^	15.4 (0.38)^d^
BMI (kg/m^2^)	2005	17.2 (2.36)	17.6 (2.71)	21.1 (3.77)	21.2 (2.93)
	2011	17.5 (2.74)	17.5 (2.62)	20.2 (2.89)^b^	21.0 (3.03)
	2018	17.4 (2.45)	17.5 (2.61)	20.4 (2.87)^b^	21.3 (3.39)
Waist circumference (cm)	2005	62.2 (7.14)	63.3 (7.65)	75.6 (9.61)	73.2 (7.02)
	2011	60.7 (6.56)^b^	59.3 (6.42)^b^	72.2 (7.33)^b^	68.8 (6.65)^b^
	2018	60.9 (6.13)^b^	59.6 (6.56)^b^	72.2 (6.23)^b^	69.0 (7.06)^b^
**Accelerometer wear compliance**					
No. of valid days	2005	4 (4 to 4)	4 (4 to 4)	4 (3 to 4)	4 (3 to 4)
	2011	7 (6 to 7)	7 (6 to 7)	6 (5 to 7)	7 (6 to 7)
	2018	6 (6 to 7)	6 (6 to 7)	6 (5 to 7)	6 (5 to 7)
Wear time (min/day)	2005	800 (66)	789 (65)	817 (90)	820 (83)
	2011	800 (62)	802 (68)^b^	833 (69)	838 (70)^b^
	2018	792 (66)	794 (66)	793 (87)^d^	824 (77)^c^

Background characteristics and wear times are mean (SD), No. of valid days are medians (25th and 75th percentiles).

BMI, body mass index.

a *n* varies for the different anthropometric measurements and is lowest (in parenthesis) for waist circumference (WC) in all age-sex groups.

b Different from 2005 (*P *≤0.04).

c Different from 2011 (*P *=* *0.03)

d Different from 2005 and 2011 (*P *≤0.01).

### Secular changes in sedentary time overall

Nine-year-old boys and girls spent an average of about 7 h 45 min sedentary per day in 2018 (Table 2). In boys, this represented an increase from both 2005 at 29 min/day (95% CI: 19, 39; *P *<0.001) and 2011 at 17 min/day (95% CI: 9, 26; *P *<0.001), with increases observed on both weekdays and weekends. The mean difference in ST observed between 2005 and 2018 in 9-year-old girls was much smaller (6 min/day), and the 95% confidence interval included unity (95% CI: -3, 16).

**Table 2:** Secular changes in sedentary time (min per day/min per segment per day) from 2005 to 2011 and 2018 (*n* = 5168^a^)

9-year-olds	Year	BoysSedentary time	Difference from 2005	Girls Sedentary time	Difference from 2005
Weekly	2005	436 (429, 443)	–	458 (451, 465)	–
	2011	448 (441, 454)	12 (2, 21)	465 (459, 471)	7 (-2, 17)
	2018	465 (458, 472)*	29 (19, 39)	464 (459, 470)	6 (-3, 16)
Weekdays	2005	444 (435, 452)	–	472 (465, 480)	
	2011	461 (454, 467)	17 (6, 27)	484 (478, 491)	11 (2, 21)
	2018	475 (468, 482)*	31 (5, 24)	480 (475, 486)	7 (-2, 17)
Weekend days	2005	419 (412, 427)	–	428 (421, 436)	–
	2011	426 (419, 433)	7 (-4, 17)	430 (423, 438)	2 (-8, 12)
	2018	450 (441, 459)*	31 (13, 36)	438 (430, 446)	9 (-2, 21)
Morning^b^	2005	74 (72, 76)	–	78 (77, 80)	–
	2011	77 (75, 78)	2 (-0,5)	79 (77, 81)	1 (-2, 3)
	2018	77 (75, 79)	3 (0,6)	79 (76, 81)	0 (-3, 3)
School^c^	2005	118 (114, 122)	–	134 (130, 138)	–
	2011	124 (121, 127)	6 (1, 11)	137 (134, 140)	3 (-2, 8)
	2018	123 (120, 127)	5 (-0, 11)	135 (132, 137)	1 (-4, 5)
After school^c^	2005	263 (257, 268)	–	272 (267, 277)	–
	2011	269 (265, 274)	7 (-1, 14)	279 (275, 283)	7 (1, 13)
	2018	283 (278, 288)*	21 (13, 28)	277 (273, 281)	5 (-1, 12)
**15-year-olds**					
Weekly	2005	555 (546, 565)	–	575 (566, 584)	–
	2011	561 (553, 570)	6 (-8, 21)	600 (593, 607)	25 (11, 38)
	2018	576 (568, 583)*	21 (8, 34)	598 (591, 605)	22 (10, 35)
Weekdays	2005	577 (567, 588)	–	607 (596, 617)	–
	2011	589 (581, 598)	13 (-2, 27)	634 (627, 642)	28 (14, 41)
	2018	605 (597, 613)*	28 (15, 41)	631 (624, 638)	25 (13, 36)
Weekend days	2005	508 (499, 517)		515 (510, 521)	–
	2011	507 (498, 517)	−1 (-15, 12)	533 (526, 541)	18 (8, 28)
	2018	519 (509, 528)	10 (-3, 23)	536 (527, 544)	21 (10, 31)
Morning^b^	2005	82 (79, 84)	–	86 (83, 88)	–
	2011	82 (79, 85)	1 (-3, 5)	89 (86, 91)	3 (-0, 7)
	2018	87 (85, 88)^d^	5 (2, 8)	90 (89, 92)	5 (2, 8)
School^c^	2005	192 (187, 197)	–	210 (203, 216)	–
	2011	198 (194, 202)	6 (-0, 13)	222 (218, 226)	13 (5, 20)
	2018	206 (202, 210)^d^	14 (8, 21)	223 (219, 227)	14 (6, 21)
After school^c^	2005	338 (330, 345)	–	339 (332, 345)	–
	2011	342 (337, 348)	5 (-5, 15)	353 (348, 357)	14 (5, 23)
	2018	349 (343, 355)	11 (2, 20)	349 (344, 354)	10 (3, 18)

Sedentary times are marginal means (95% CI) adjusted for age, wear time, seasonality (daylight) and weekday-weekend day ratio (weekly values only), with 95% CIs adjusted for school-level cluster sampling.

a *n* varies from 579 (boys, 2005) to 474 (boys, 2011) in 9-year-olds and from 410 (girls, 2018) to 296 (boys, 2011) in 15-year-olds for the analyses of weekly, weekday, weekend day, school and after-school sedentary time, but is lower in analyses of morning sedentary time (between 481 (girls, 2018) and 335 (girls, 2005) in 9-year-olds and between 309 (girls, 2018) and 178 (boys, 2005).

b 06:00–09:00.

c 9:00–13:00 and 13:00-22:00 for 9-year-olds, 09:00–14:00 and 14:00–23:00 for 15-year-olds.

d Sedentary time in 2018 different from 2011 (*P *≤0.015).

In 15-year-olds, ST increased overall among boys and girls between 2005 and 2018, by 21 min/day (95% CI: 8, 34; *P *=* *0.002) to an average of 9 h 36 min/day in boys, and in girls by 22 min/day (95% CI: 10, 35; *P *<0.001) to an average of 9 h 58 min/day (Table 2). In boys, the change was gradual between 2005, 2011 and 2018 and was more pronounced on weekdays at 28 min/day (95% CI: 15, 41; *P *<0.001) than on weekend days at 10 min/day (95% CI: -3, 23; *P *=* *0.124). In girls, the increase in ST was similar on weekdays and weekend days between 2005 and 2011, with no further increase observed between 2011 and 2018 overall at -2 min/day (95% CI: -11, 7; *P *=* *0.660).

**Figure 2:**
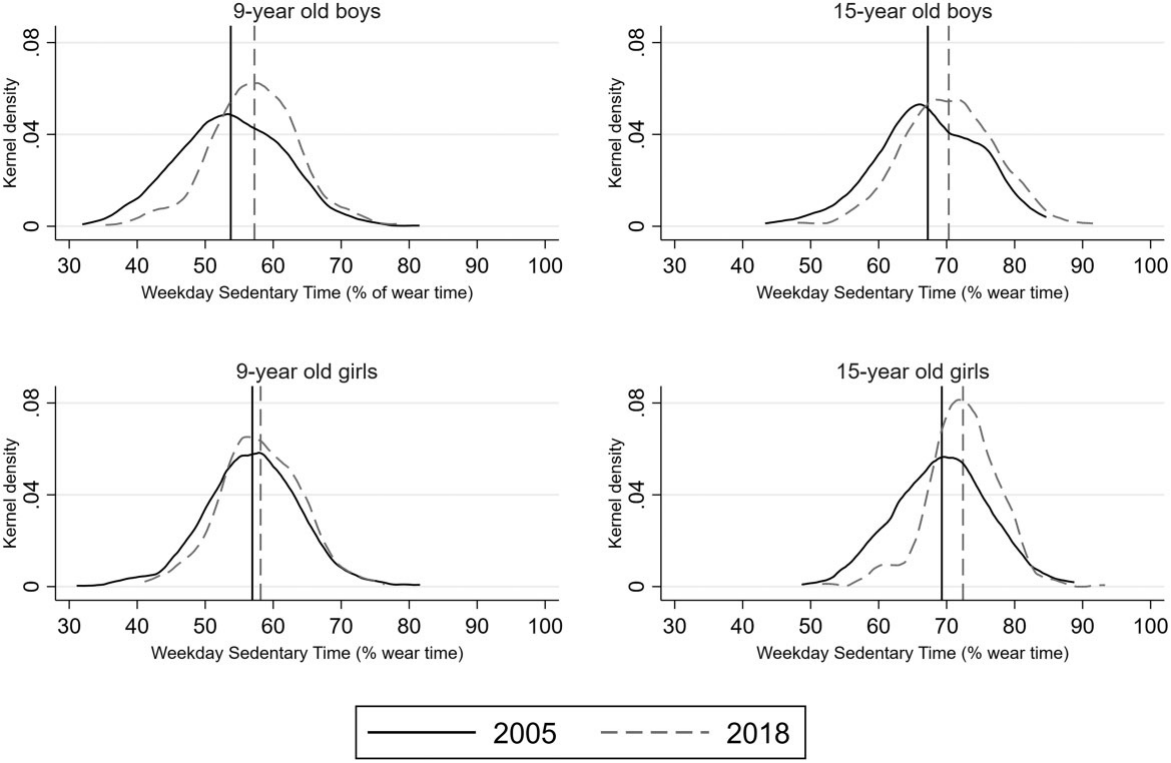
Secular change in the distribution of total weekday sedentary time between 2005 and 2018. Data are normalized to total weekday wear-time. The distribution is not adjusted for age or minutes of daylight. Vertical lines are survey medians

Density plots suggest that secular changes in ST from 2005 to 2018 are attributable to a right-shift of the distribution (see weekday total ST in Figure 2, and weekend total ST in the Supplementary Material, page 4). However, in 9-year-old boys and 15-year-old girls, a change in the shape of the distribution has also contributed.

### Secular changes in bouts of sedentary time

**Figure 3:**
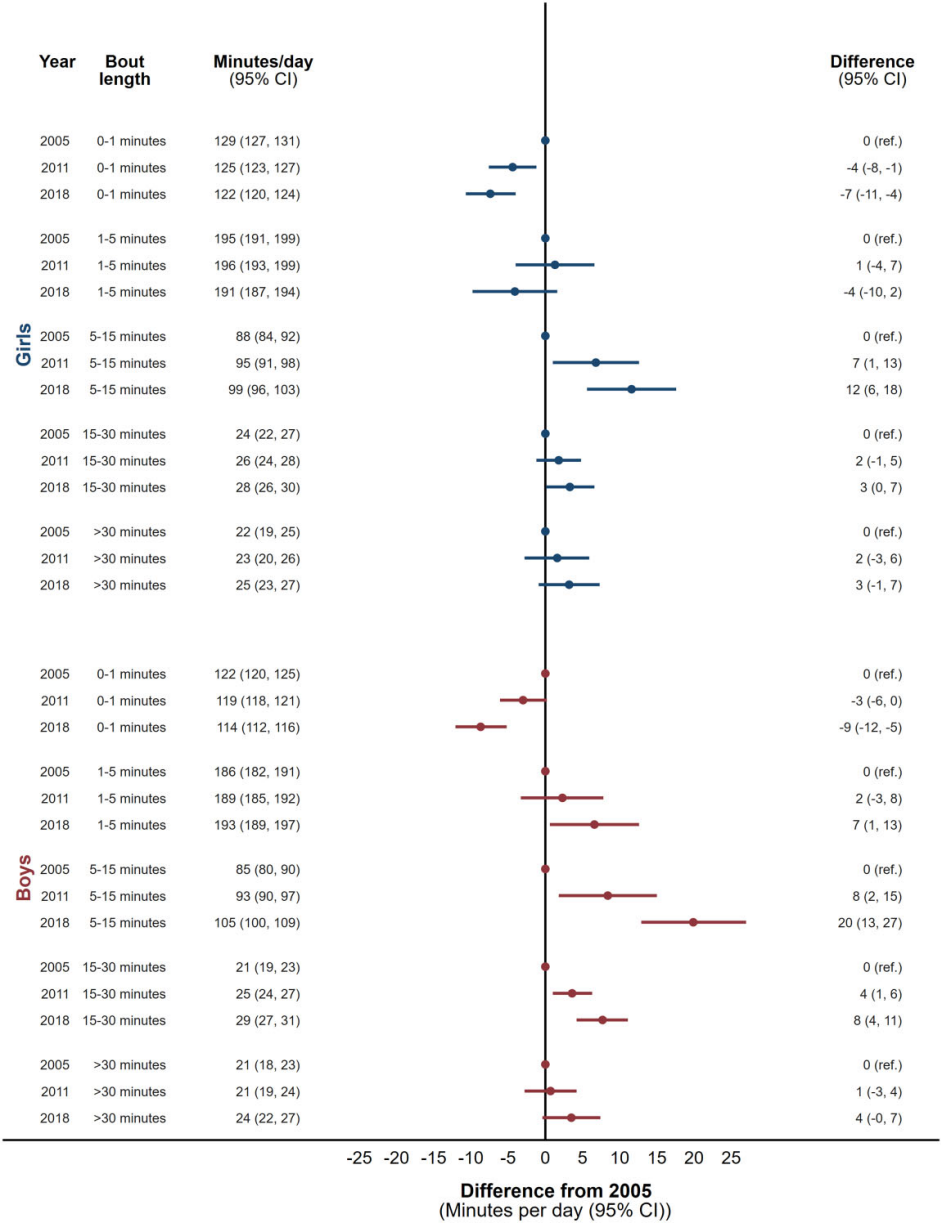
Secular trends in sedentary time (min per day) accumulated in uninterrupted bouts lasting 0 to 1 minute, 1 to 5 min, 5 to 15 min, 15 to 30 min and >30 min between 2005, 2011 and 2018 in 9-year-old girls and boys. Analyses are adjusted for age, minutes of daylight at the time of sedentary time assessment, mean daily accelerometer wear time,and the ratio of valid weekdays to weekend days

From 2005 to 2018, 9-year-olds increased ST accumulated in bouts of 5 to 15, 15 to 30 and >30 min, with the largest differences observed for bouts lasting 5 to 15 min (Figure 3). Sedentary time accumulated in very short bouts (0 to 1 min) was significantly lower in 2018 compared with 2005. Changes were larger in boys than in girls, but the pattern of change was similar with one exception: girls showed a tendency towards less ST accumulated in bouts of 1 to 5 min, whereas time in 1 to 5-min ST bouts increased significantly in boys.

**Figure 4:**
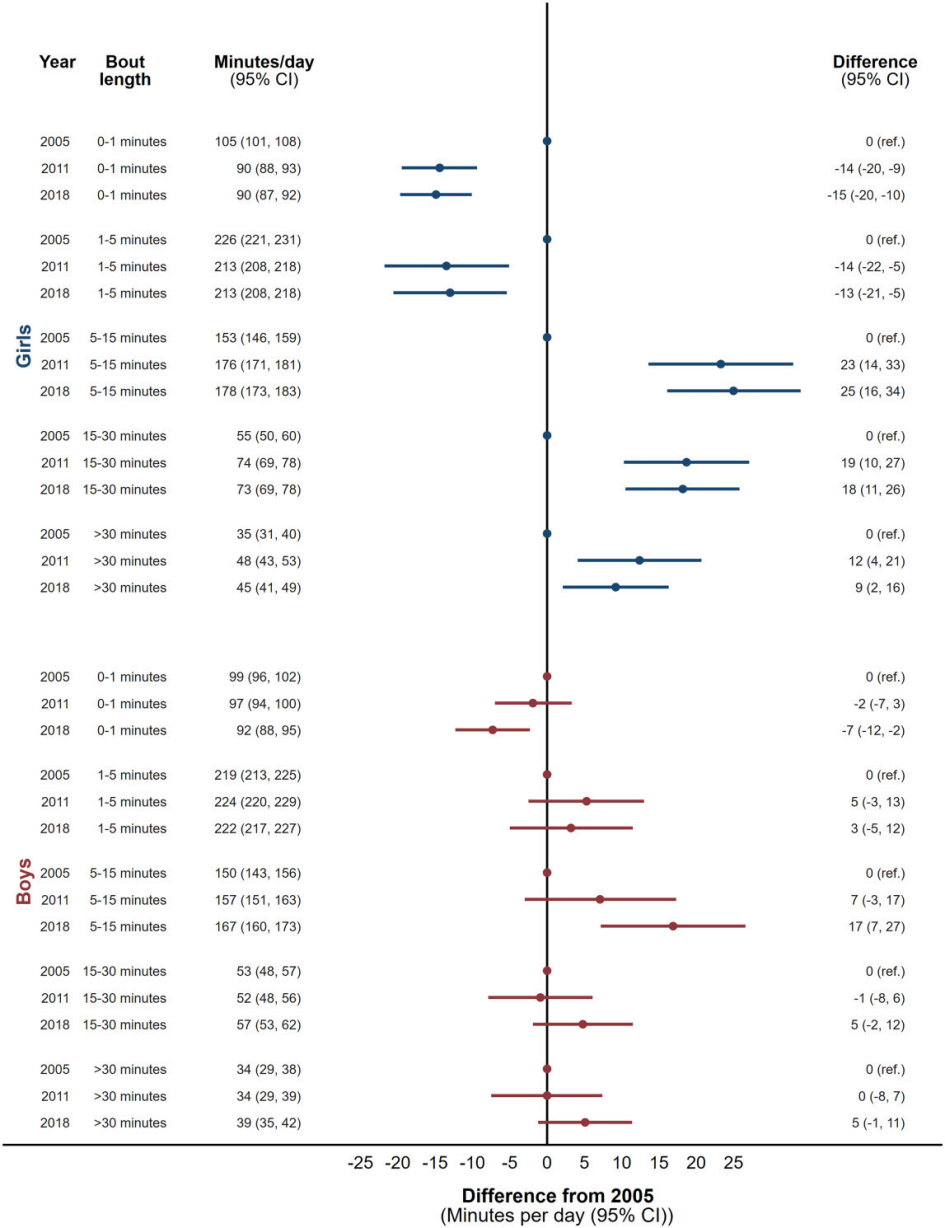
Secular trends in sedentary time (min per day) accumulated in uninterrupted bouts lasting 0 to 1 minute, 1 to 5 min, 5 to 15 min, 15 to 30 min and >30 min between 2005, 2011 and 2018 in 15-year-old girls and boys. Analyses are adjusted for age, minutes of daylight at the time of sedentary time assessment, mean daily accelerometer wear time and the ratio of valid weekdays to weekend days

A similar pattern, of less time in shorter bouts replaced with more time in longer bouts of ST, was observed in 15-year-old boys and girls (Figure 4). Adolescents girls showed larger absolute secular changes than adolescent boys, particularly of 5 to 15-min duration. Some changes in bout durations among adolescent boys had 95% confidence intervals including unity.

Both boys and girls of both age groups showed identical patterns on weekdays and weekend days, but with more pronounced changes on weekdays (Supplementary Material, pages 5-8). Broadly speaking, this same pattern was also observed when breaking down weekdays into morning, school day and after school segments (Supplementary Material, pages 9-14).

## Discussion

We observed an increase of 20 to 30 min/day of device-measured ST in 9-year-old boys and in 15-year-old boys and girls from 2005 to 2018. All age-sex groups spent less ST in bouts lasting <5 min and more time in longer bouts of uninterrupted ST, particularly those of 5 to 15-min duration. Adolescent girls also had a substantial increase in ST accumulated in bouts lasting 15 to 30 min and 30 min or more. These trends were largely mirrored across weekdays, weekends and weekday-segments, suggesting that secular changes in ST are not explained by sedentary behaviours during specific periods of the week. Our data suggest that contemporary children and adolescents are spending a larger part of their day in uninterrupted sedentary pursuits than before the introduction of smartphones, tablets and near-universal internet access.

We observed a right-shift of the ST distribution, with an exception among 9-year-old girls. Whereas this is consistent with a similar increase at all levels of ST, the reduced variance (tighter bell-shape) suggests that secular increases have been larger at the lower end of the distribution in 9-year-old boys and 15-year-old girls. Hence, in these groups, fewer children and adolescents are now at the lower end of the ST distribution. We did not have access to comparable information on parental education or income across surveys, which prohibits us from examining secular changes across socioeconomic gradients.

Device-based measurements of ST in large epidemiological studies have become feasible within the past two decades. Yet, such data are only available in a few population-based surveys and even fewer have data covering multiple cycles, a prerequisite of monitoring secular trends.^10^ The Canadian Health Measures Survey assessed ST using devices during five cross-sectional cycles from 2007 to 2017.^9^ Between their samples, total ST decreased from just over 9 h/day in the 2007-09 to less than 9 h/day in 2016-17 in 12 to 17-year-olds, and remained stable at about 7.5 hrs/day in 6 to 11-year-olds.^9^ Hence, Canadian data are not in agreement with our data, which may reflect true differences in trends between the two countries. Importantly, stable total ST does not preclude changes in the ST accumulation pattern. This is illustrated by our data in 9-year-old girls, who saw no change in total ST from 2005 to 2018, yet replaced ST accumulated in short bouts with more time spent in longer uninterrupted ST. It is also noteworthy that whereas changes in total ST were broadly similar in 15-year-old girls and boys, changes in accumulation patterns were more pronounced in girls.

Self-reported ST from the National Health and Nutrition Examination Survey (NHANES), operationalized as total sitting time, suggests an increase in US adolescents’ ST between 2007 and 2016 of 1.1 h/day (95% CI: 0.7, 1.5).^23^ However, self-reported ST is prone to recall and social desirability biases and shows poor agreement with device-measured data.^24^ Yet, self-reported measures of sedentary behaviours remain an important tool in epidemiological studies for determining place and context. For example, changes in ST among US adolescents were attributed to more computer use while television viewing had remained stable.^23^ In the European arm of the HBSC survey covering 2002 to 2014, an increase in adolescents’ computer use coincided with less television viewing,^8^ exemplifying the challenges of studying secular trends in the total volume of ST based on measures of ever-evolving screen-based leisure-time pursuits. Also, the nature of the survey item suggests that increased computer use in the HBSC may also reflect displacement of homework to computers, which would not impact total ST, further highlighting the challenges of monitoring population trends in ST based on self-reported data.

It is difficult to establish if an average increase in ST of 20 to 30 min/day in children and adolescents is a cause for concern. Currently no quantitative target is given for total ST, and the health impacts of more ST is likely determined by the behaviour that is being replaced, with more evidence to support adverse consequences of less time spent in moderate-to-vigorous physical activity.^1^ For example, in a large cross-sectional analysis of more than 10 000 adolescents from several countries, isotemporal substitution modelling suggested that replacing 10 min/day of ST with light-intensity physical activity was associated with 0.05 cm lower waist circumference, whereas the same substitution with moderate-to-vigorous physical activity was associated with a 10-fold larger effect size of 0.6 cm.^25^ If these associations are causal, an increase in daily ST of 20 to 30 min would not be trivial.

Currently, the combined evidence from literature reviews prepared for the 2020 WHO Physical Activity Guideline Development Group suggested that children and adolescents should limit ST in general, and particularly ST accumulated from recreational screen-time.^1^ From our data, we cannot determine if changes in ST reflect additional screen or non-screen behaviours, even if ecological-level evidence suggests smartphones or tablets are plausible contributors.^6–8^^,^^26^^,^^27^ More time allocated to computer use is also a likely contributor.^8^^,^^23^ On the other hand, increased mobility and gamification through screen-based devices may also open relevant opportunities for physical activity for some individuals.^28^ Despite calls for coordinated efforts to decrease physical inactivity,^29^ global prevalence estimates of physical inactivity among children and adolescents suggest little change since the start of the century,^30^ and in Norway the prevalence of inactive 9-year-olds has increased.^12^ This is consistent with a general increase in the availability of attractive sedentary pursuits across the day. With technological developments and further integration of devices and online platforms into everyday life, physical activity and ST surveys need to be developed so that these instruments can assess changes in human physical activity behaviour now and in the future.

It is insufficiently understood whether prolonged sedentary bouts are a particular health risk in children and adolescents, above and beyond the total volume of ST.^4^ In observational studies, prolonged sedentary bouts may reflect engagement in specific sedentary behaviours such as watching television or gaming. Hence, it is possible that other behaviours during engagement in these activities, such as snacking, are the causative factors leading to stronger associations between prolonged bouts of ST and health outcomes. Additional research is needed to understand the consequences of specific ST accumulation patterns on health-related outcomes.

We highlight the following limitations. The PANCS surveys do not have sampling weights available to account for a potential systematic bias in non-participation across socioeconomic or geographical characteristics. Similarly, we do not have access to data allowing a formal comparison of our samples with characteristics of the general population. Registry-based parental education was available in PANCS2, suggesting no socioeconomic bias.^12^ Participation rates were lower in 2011 and 2018 compared with 2005, which could bias secular trends if non-participation is associated with levels or patterns of ST. Participation in health surveys is often positively associated with socioeconomic factors in high-income countries,^31^ factors that have also been associated with lower levels of screen-based ST in children.^32^ Therefore, under the assumption that differences in non-participation would lead to more socioeconomically advantageous samples in 2011 and 2018, we expect the direction of bias would lead to an underestimation of secular changes in ST from 2005. However, we note that differences between participants and non-participants would have to be large to make a qualitative difference to an already substantial change of 20 to 30 min ST/day.

Hence, we believe our main message of increases in ST over the past 12 years is robust to potential non-identical participation mechanisms across surveys. Also, the participation rate among 9-year-olds can be considered high across surveys. Hip-worn accelerometers are unable to discern sitting, lying or reclining from standing which may lead to some misclassification. This would bias estimates of secular changes in ST if standing behaviours have changed.^33^ Finally, we did not use the same ActiGraph accelerometer model across surveys. Results of small, free-living and mechanical validations,^14^ including our own analysis of absolute sedentary bout agreement (Supplementary Material, page 2), suggest this potential source of error is very small following LFE filtering. The different trends observed in boys and girls, and on week and weekend days, also suggest that a systematic inter-model measurement error is unlikely.

## Conclusion

In conclusion, from 2005 to 2018, ST has increased by almost 30 min/day in 9-year-old boys, has been stable in 9-year-old girls, and has increased by about 20 min/day in 15-year-old boys and girls from Norway. Children and adolescents accumulate more of their ST in bouts lasting longer than 5 min than they did before the introduction of smartphones and ubiquitous internet access.

## Ethics approval

The Regional Committee for Medical Research Ethics (RCMRE) approved PANCS1 (REK Sør: S-04305), whereas PANCS2 (REK Sør-Øst: 2010/3127) and PANCS3 (REK Sør-Øst: 2017/1426) were considered outside the Health Research Act's scope by the RCMRE and therefore not considered subject to approval. The Norwegian Centre for Research Data approved the processing of personal data in all three study waves (ref. numbers 12166, 25870 and 54951).^11^

## Data availability

The approval from the Ethics Committee does not include permission to make data materials publicly available. Analytical code (Stata do-files) will be made available upon request.

## Supplementary data

Supplementary data are available at *IJE* online.

## Author contributions

K.E.D. (guarantor) and J.T. had the idea for and designed the study, pooled and linked the data, analysed the data with help from J.S.S., B.H.H. and EK and take responsibility for its integrity and the data analysis. K.E.D. and J.T. wrote the first draft of the report. All other authors assisted in developing the statistical models, reviewed results, provided guidance on methods and critically reviewed the manuscript. All authors read and approved the final version of the manuscript.

## Funding

This work was supported by the Norwegian Research Council (grant number 249932/F20) and by the Department of Sports Medicine, the Norwegian School of Sport Sciences.

## Supplementary Material

dyac063_Supplementary_Data
