# Supplementary material for: Device-measured sedentary time in Norwegian children and adolescents in the era of ubiquitous internet access: secular changes between 2005, 2011 and 2018
Source: Int J Epidemiol. 2022 Apr 1;51(5):1556–67. doi: 10.1093/ije/dyac063 (PMC9557841; doi:10.1093/ije/dyac063)
Supplement: dyac063_Supplementary_Data [file dyac063_supplementary_data.pdf]

Supplementary material

Supplement to: Dalene KE, Kolle E, Steene-Johannessen J, et al. Device-measured sedentary time in Norwegian children and adolescents in the era of ubiquitous internet access: Secular changes between 2005, 2011 and 2018

Supplemental Figure 1. Analysis of sedentary time and sedentary bouts using different Actigraph monitors and filtering

Supplementary Table 1. Accelerometer data inclusion criteria

Supplemental Figure 2. Crude distribution of sedentary time on weekend days by age-group and sex

Supplemental Figure 3. Weekday sedentary time accumulation patterns in 9-year old boys and girls

Supplemental Figure 4. Weekday sedentary time accumulation patterns in 15-year old boys and girls

Supplemental Figure 5. Weekend sedentary time accumulation patterns in 9-year old boys and girls

Supplemental Figure 6. Weekend sedentary time accumulation patterns in 15-year old boys and girls

Supplemental Figure 7. Morning sedentary time accumulation patterns in 9-year old boys and girls

Supplemental Figure 8. Morning sedentary time accumulation patterns in 15-year old boys and girls

Supplemental Figure 9. School sedentary time accumulation patterns in 9-year old boys and girls

Supplemental Figure 10. School sedentary time accumulation patterns in 15-year old boys and girls

Supplemental Figure 11. Afterschool sedentary time accumulation patterns in 9-year old boys and girls

Supplemental Figure 12. Afterschool sedentary time accumulation patterns in 15-year old boys and girls

Supplemental Figure 1. Analysis of sedentary time and sedentary bouts using different Actigraph monitors and filtering

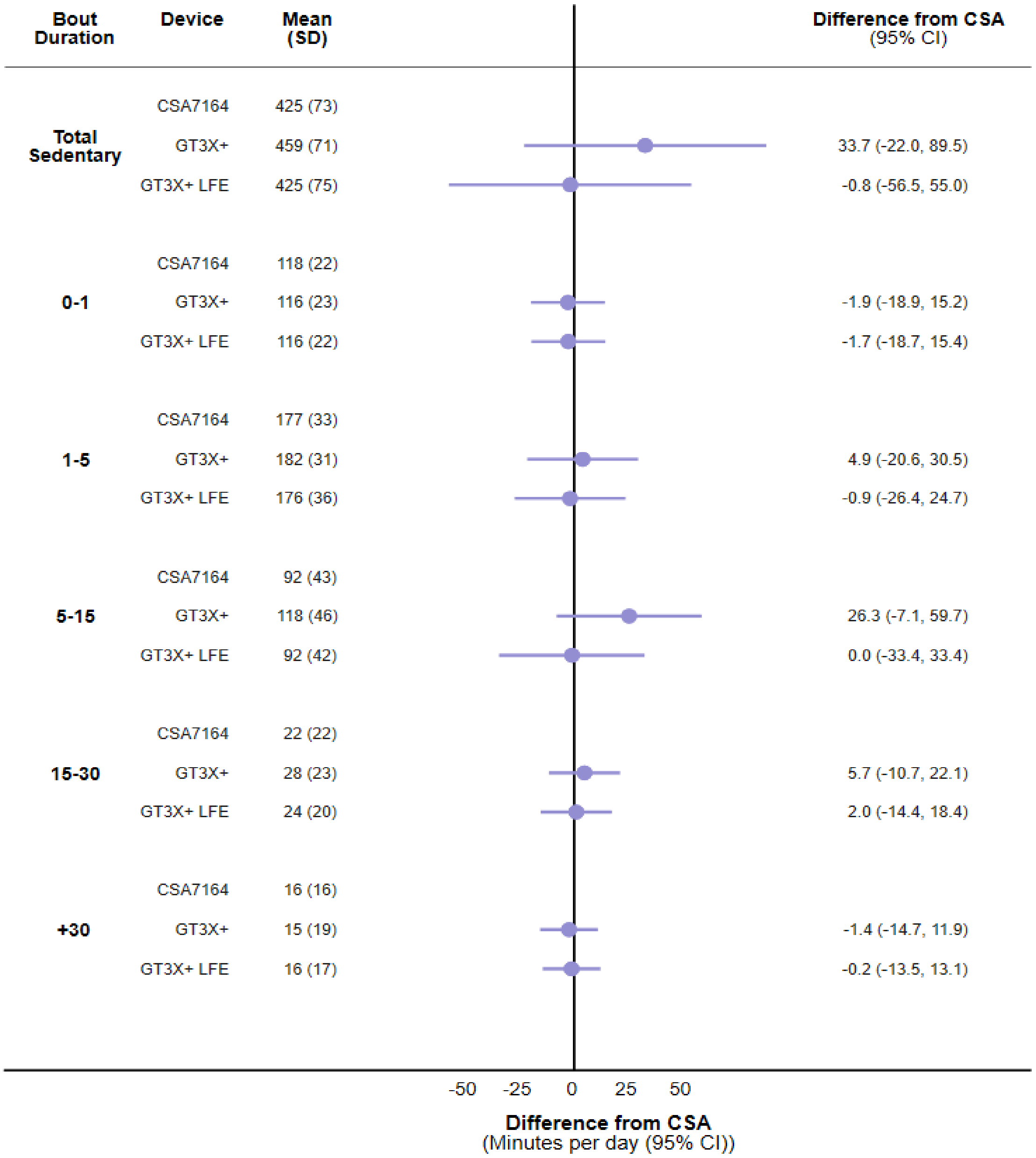

N = 14. Data was collected for three days in ten-year old Norwegian children as described in Grydeland et al. (2014).[1] Data-reduction was performed as detailed in the present manuscript. Actigraph CSA7161 monitors were used in PANCS1. Versions of the GT3X+ were used in PANCS2 and PANCS3. Data from GT3X+ monitors were analysed with (GT3X+ LFE) and without (GT3X+) activating the low-frequency extension (LFE) filter option. Using the LFE results in almost complete attenuation of inter-generation differences in sedentary time.

Supplementary Table 1. Accelerometer data inclusion criteria

		Time	Valid day/segment (% of day/segment)	Included if # of valid days/segments:
Weekdays		06:00:00-00:00:00	≥480 minutes (44%)	≥1
Weekend days		06:00:00-00:00:00	≥480 minutes (44%)	≥1
School day segments				
	Morning	06:00:00-08:59:50	≥90 minutes (50%)	≥1
	School 9-year olds	09:00:00-13:00:00	≥180 minutes (75%)	≥1
	Afterschool 9-year olds	13:00:10-22:00:00	≥270 minutes (50%)	≥1
	School 15-year olds	09:00:00-14:00:00	≥240 minutes (75%)	≥1
	Afterschool 15-year olds	14:00:10-23:00:00	≥270 minutes (50%)	≥1

Supplemental Figure 2. Crude distribution of sedentary time on weekend days by age-group and sex

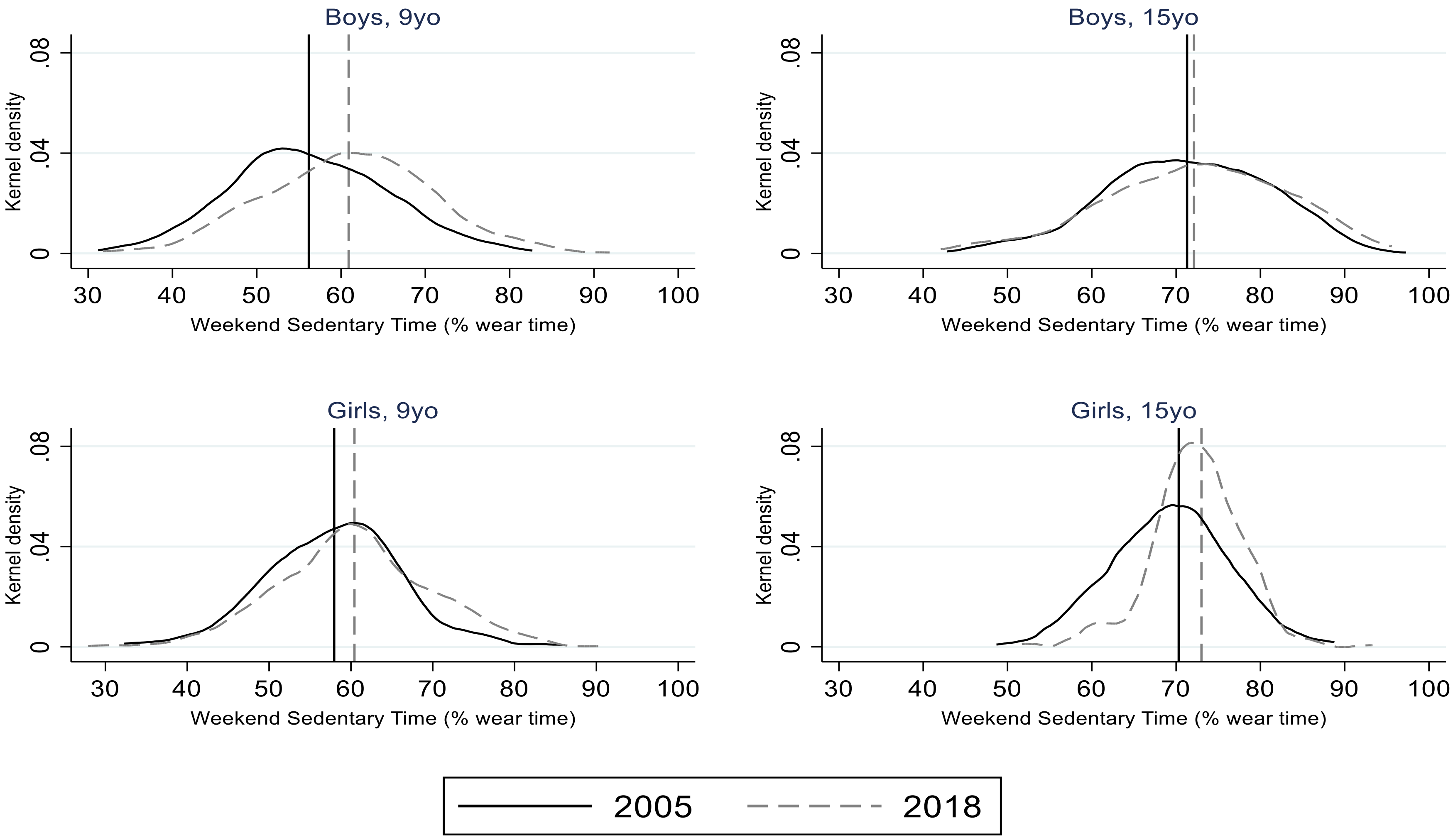

Data are normalized to total weekend wear-time. The distribution is not adjusted for age or minutes of daylight. Vertical lines are survey medians.

Supplemental Figure 3. Weekday sedentary time accumulation patterns in 9-year old boys and girls

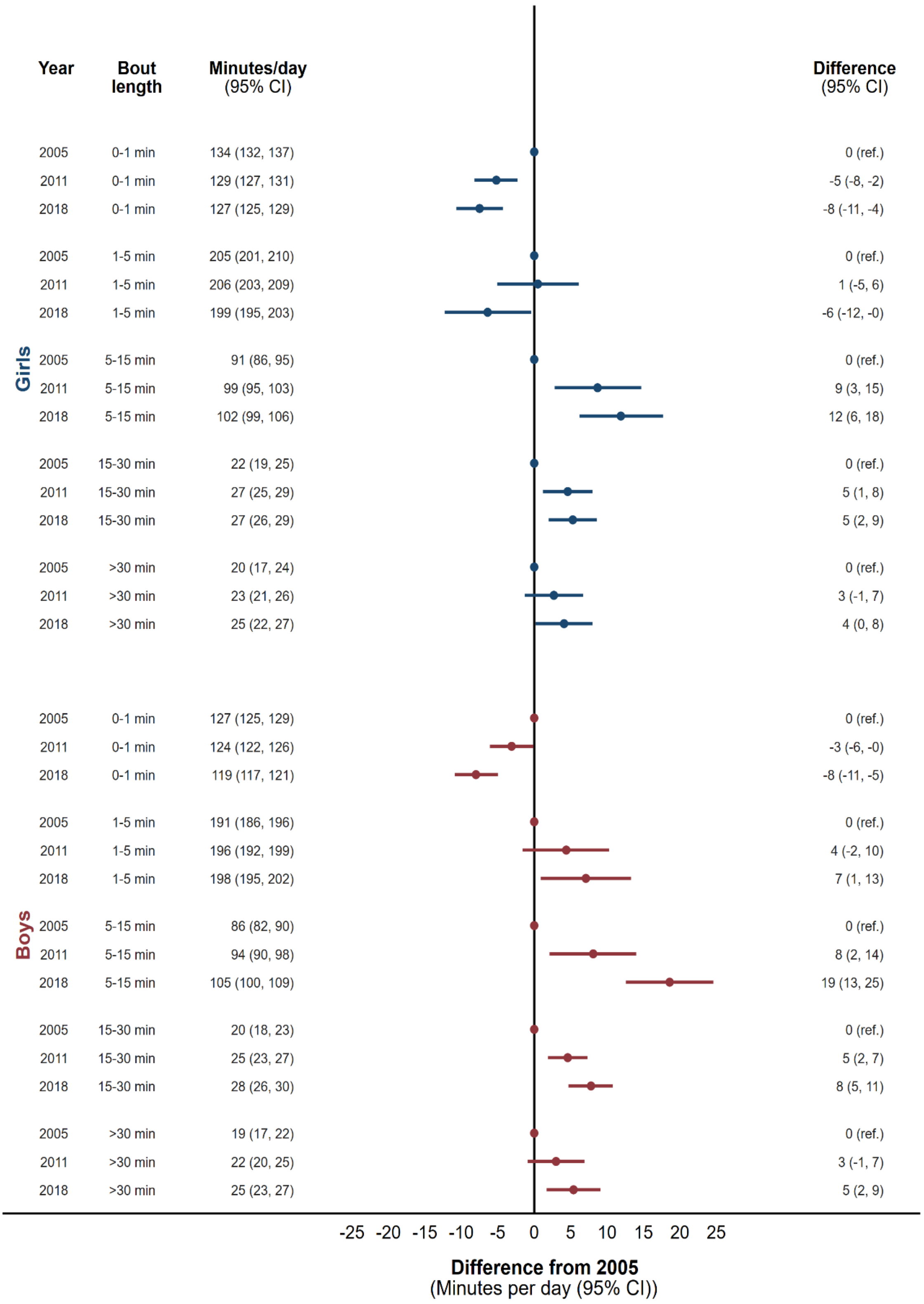

Adjusted for age, minutes of daylight, and weekday wear-time.

Supplemental Figure 4. Weekday sedentary time accumulation patterns in 15-year old boys and girls

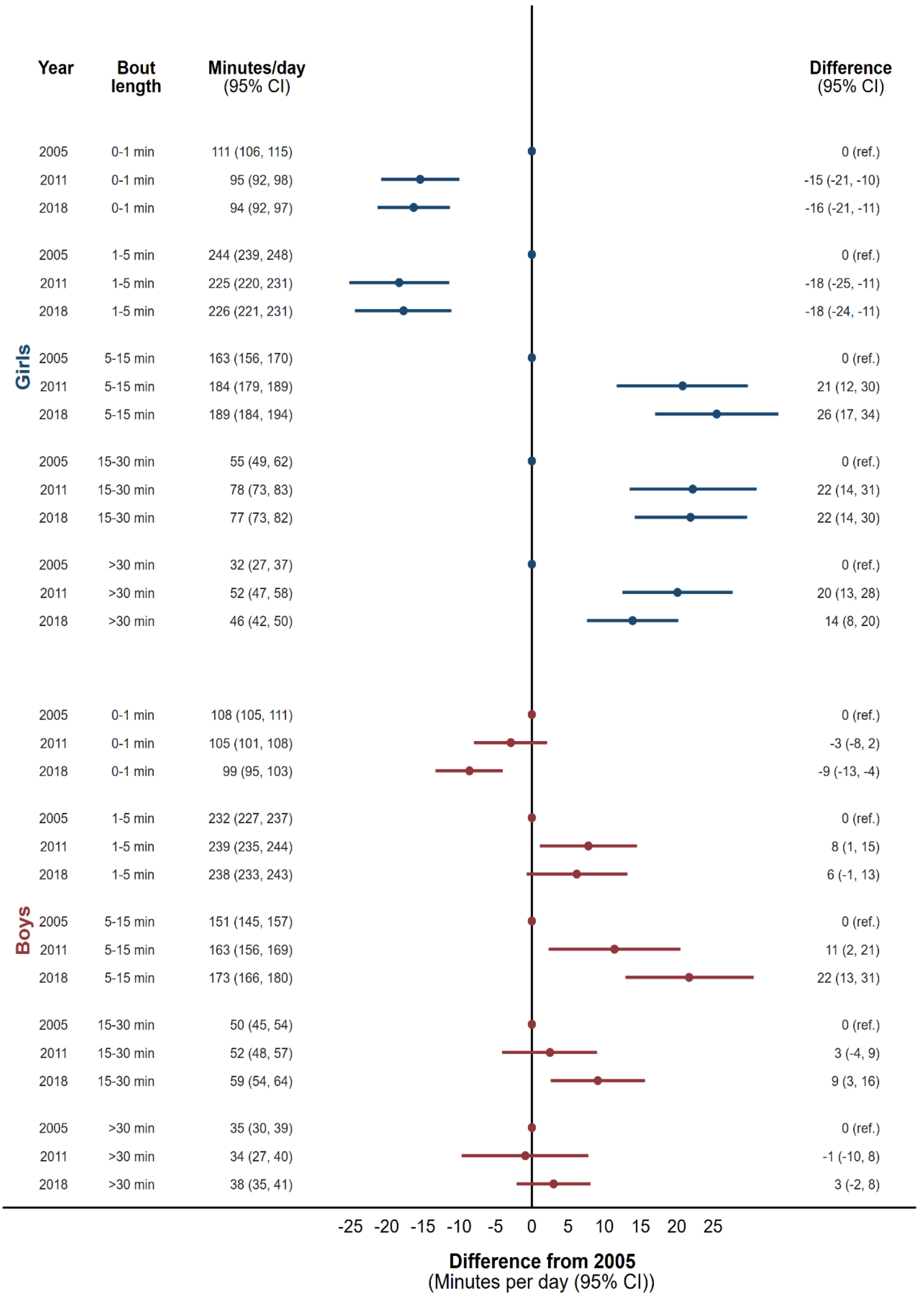

Adjusted for age, minutes of daylight, and weekday wear-time.

Supplemental Figure 5. Weekend sedentary time accumulation patterns in 9-year old boys and girls

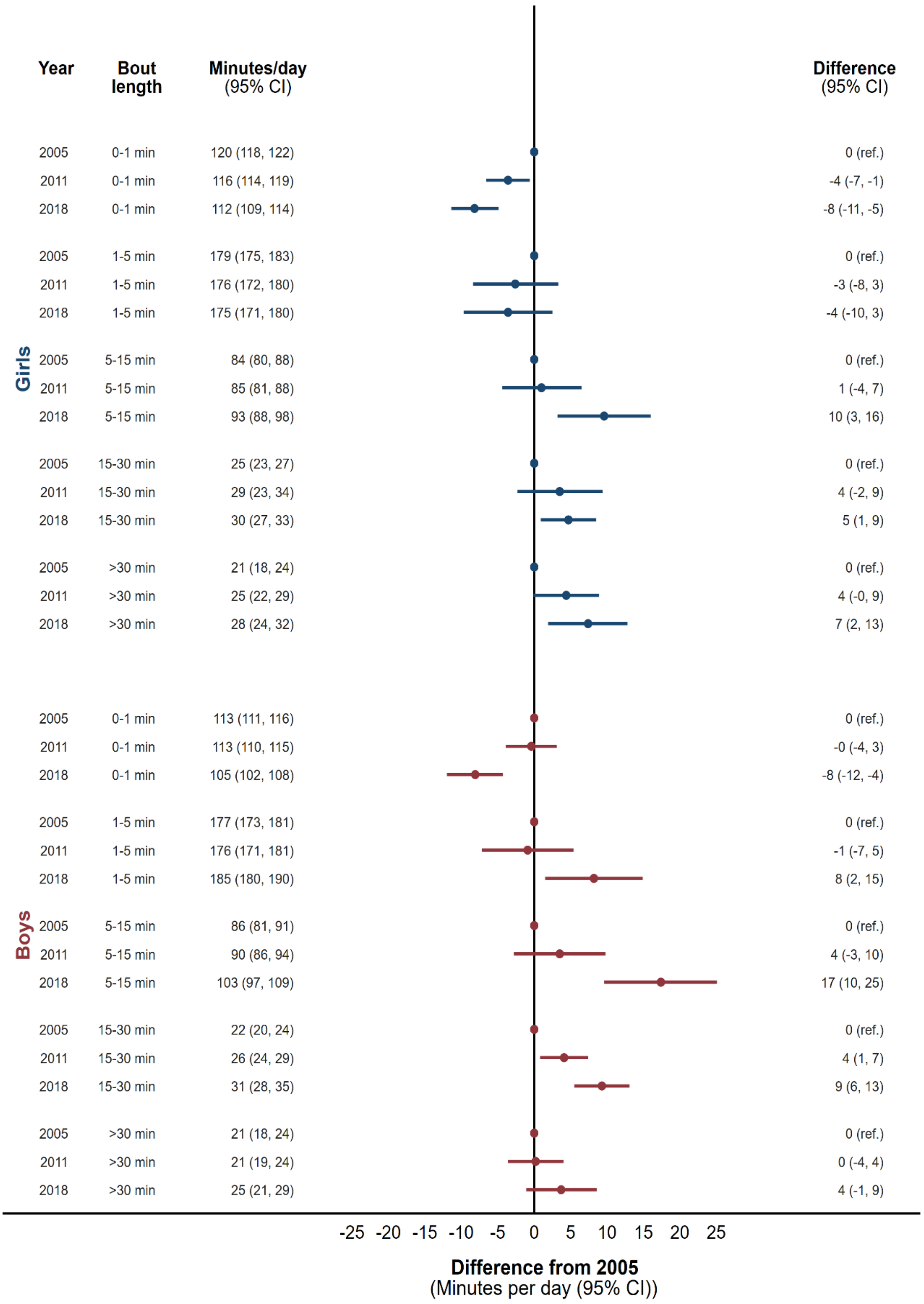

Adjusted for age, minutes of daylight, and weekend wear-time.

Supplemental Figure 6. Weekend sedentary time accumulation patterns in 15-year old boys and girls

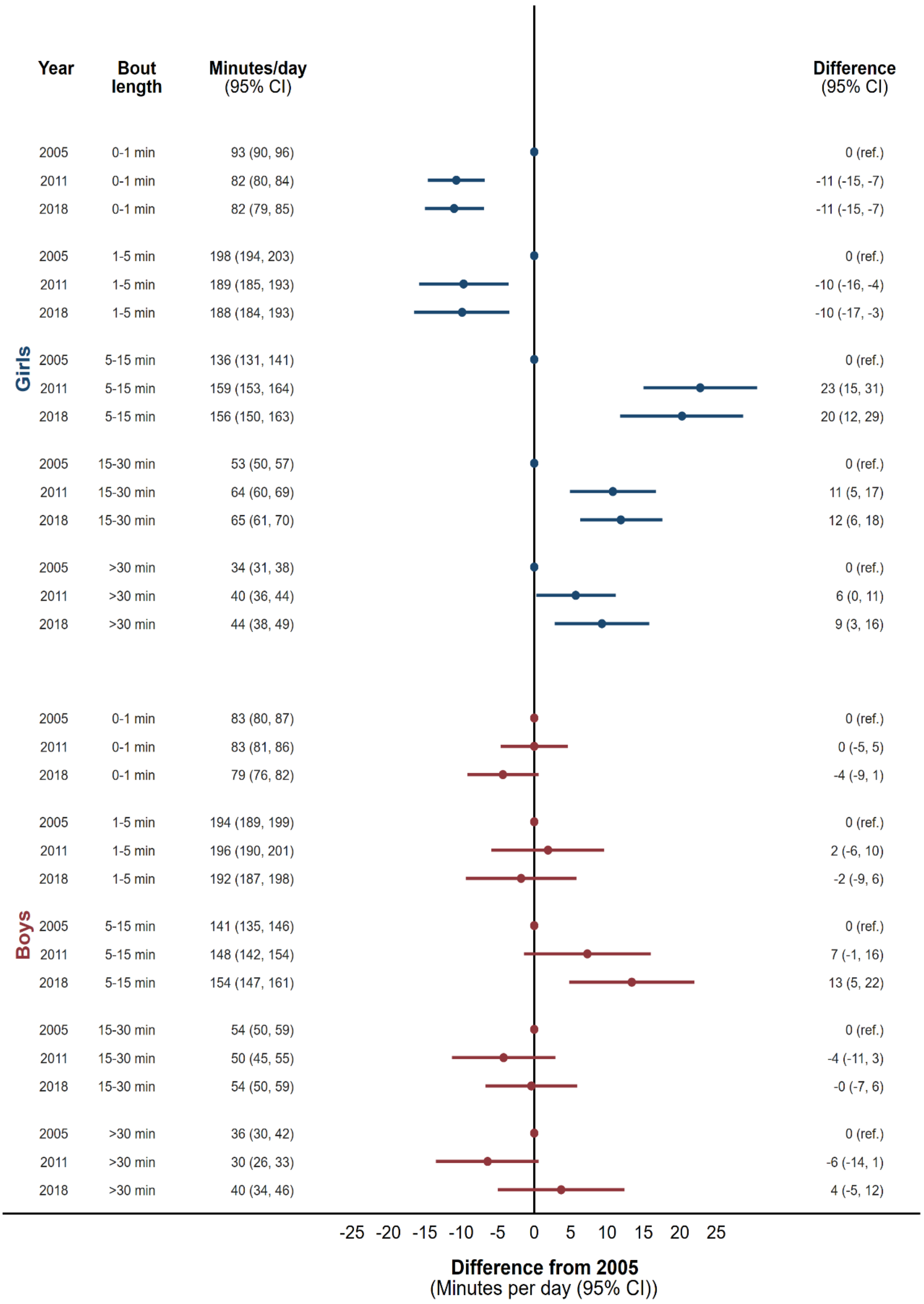

Adjusted for age, minutes of daylight, and weekend wear-time.

Supplemental Figure 7. Morning sedentary time accumulation patterns in 9-year old boys and girls

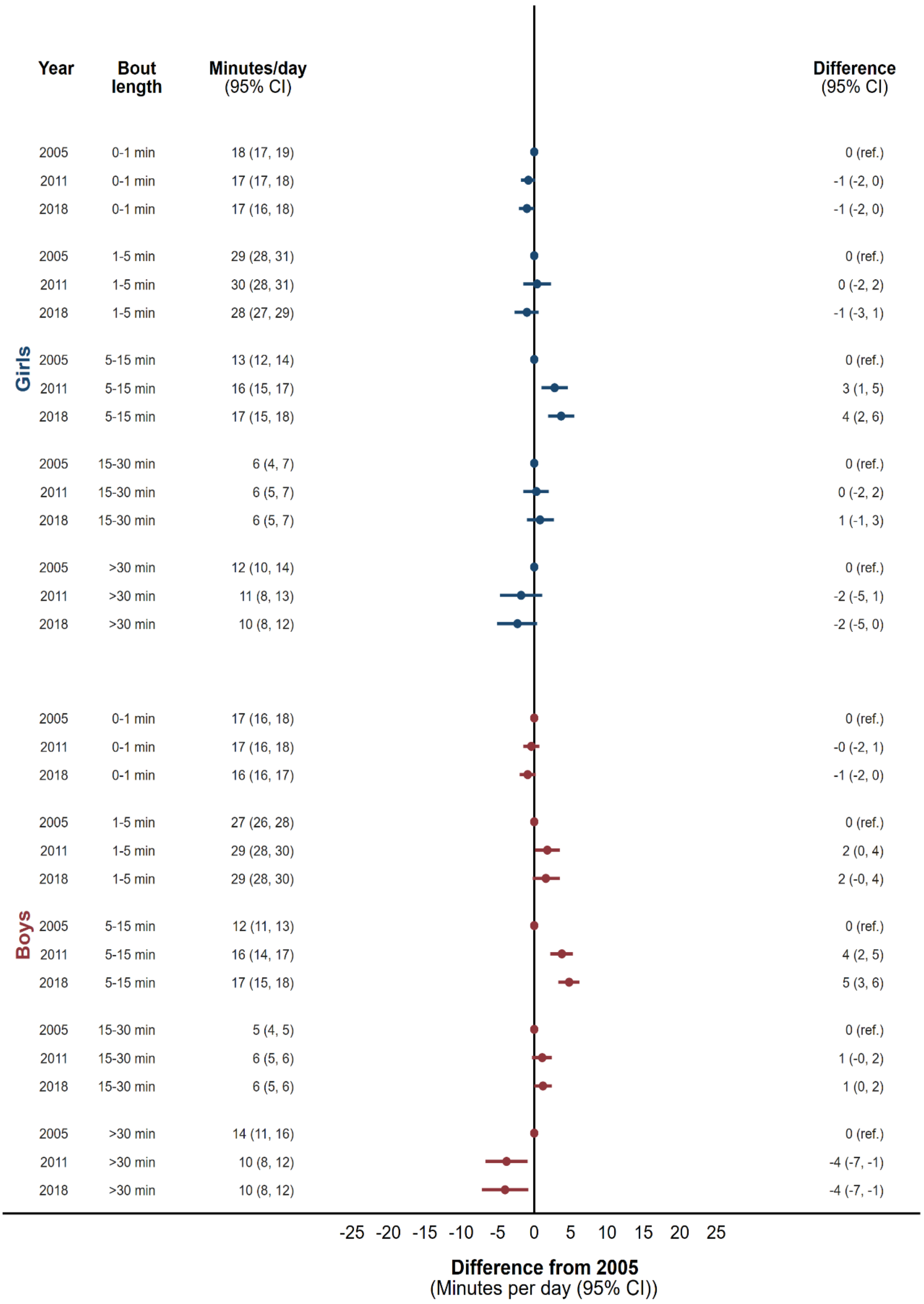

Girls; n = 1253, boys; n = 1224. Adjusted for age, minutes of daylight, and wear-time during mornings. Inclusion criteria shown in Supplemental Table 1.

Supplemental Figure 8. Morning sedentary time accumulation patterns in 15-year old boys and girls

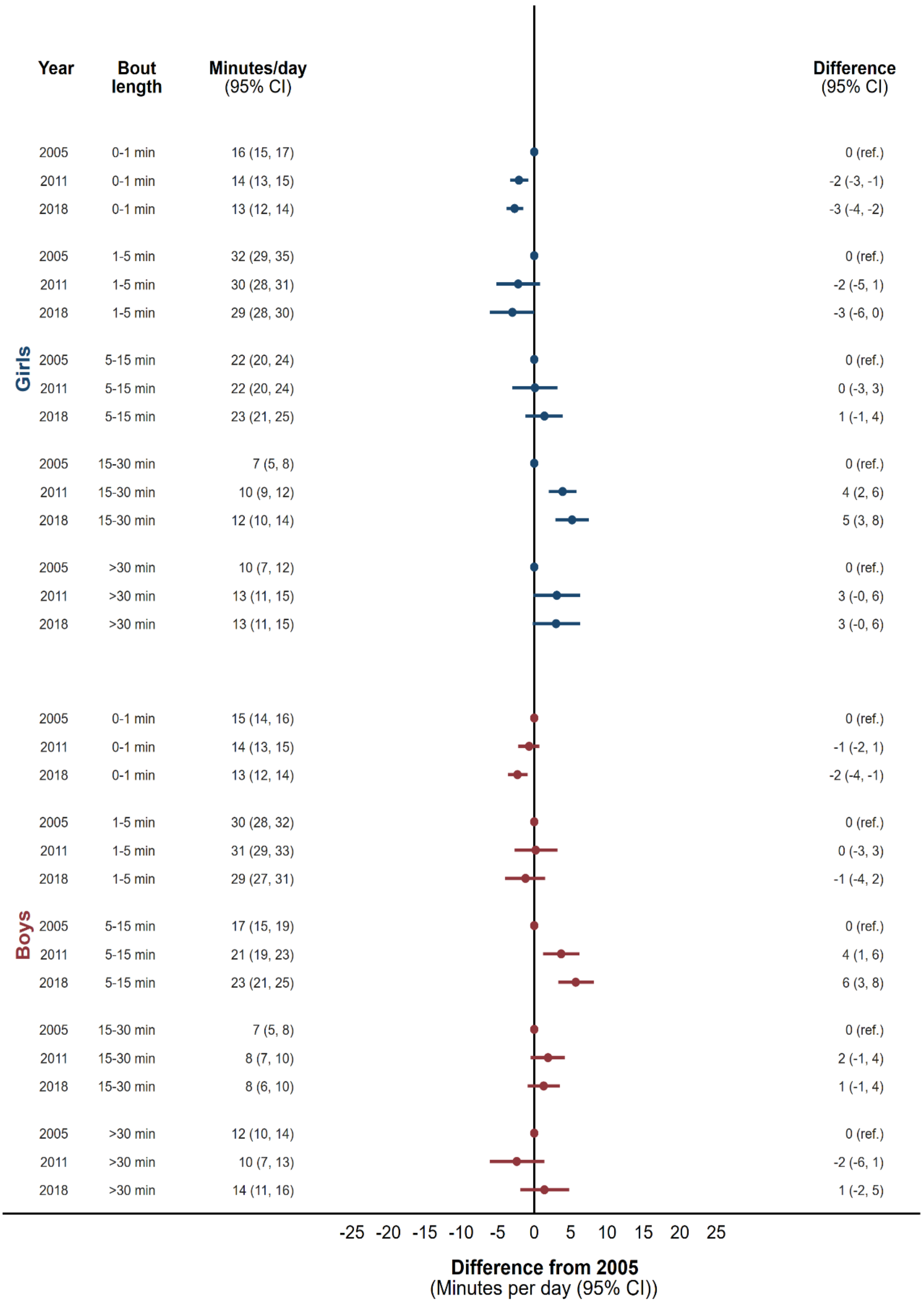

Girls; n = 787, boys; n = 607. Adjusted for age, minutes of daylight, and wear-time during mornings. Inclusion criteria shown in Supplemental Table 1.

Supplemental Figure 9. School sedentary time accumulation patterns in 9-year old boys and girls

	Year	Bout length	Minutes/day (95% CI)	Difference (95% CI)
Girls	2005	0-1 min	41 (39, 42)	0 (ref.)
	2011	0-1 min	39 (38, 40)	-2 (-3, -0)
	2018	0-1 min	39 (38, 40)	-2 (-3, -0)
	2005	1-5 min	64 (61, 67)	0 (ref.)
	2011	1-5 min	65 (63, 66)	1 (-3, 4)
	2018	1-5 min	63 (61, 64)	-1 (-5, 2)
	2005	5-15 min	25 (22, 27)	0 (ref.)
	2011	5-15 min	28 (26, 30)	3 (-0, 6)
	2018	5-15 min	27 (26, 29)	3 (-0, 5)
	2005	15-30 min	4 (3, 5)	0 (ref.)
	2011	15-30 min	4 (4, 5)	1 (-1, 2)
	2018	15-30 min	5 (4, 5)	1 (-0, 2)
	2005	>30 min	1 (1, 2)	0 (ref.)
	2011	>30 min	2 (1, 3)	1 (-0, 2)
	2018	>30 min	2 (1, 2)	1 (-0, 1)
Boys	2005	0-1 min	40 (39, 41)	0 (ref.)
	2011	0-1 min	39 (38, 40)	-2 (-3, 0)
	2018	0-1 min	38 (37, 39)	-2 (-3, -0)
	2005	1-5 min	56 (53, 59)	0 (ref.)
	2011	1-5 min	59 (57, 61)	3 (-0, 6)
	2018	1-5 min	58 (56, 60)	2 (-1, 5)
	2005	5-15 min	19 (17, 20)	0 (ref.)
	2011	5-15 min	22 (20, 24)	3 (1, 6)
	2018	5-15 min	22 (21, 24)	4 (1, 6)
	2005	15-30 min	3 (2, 3)	0 (ref.)
	2011	15-30 min	3 (3, 4)	1 (-0, 2)
	2018	15-30 min	3 (3, 4)	1 (-0, 2)
	2005	>30 min	1 (0, 1)	0 (ref.)
	2011	>30 min	1 (1, 2)	1 (-0, 2)
	2018	>30 min	2 (1, 2)	1 (-0, 2)

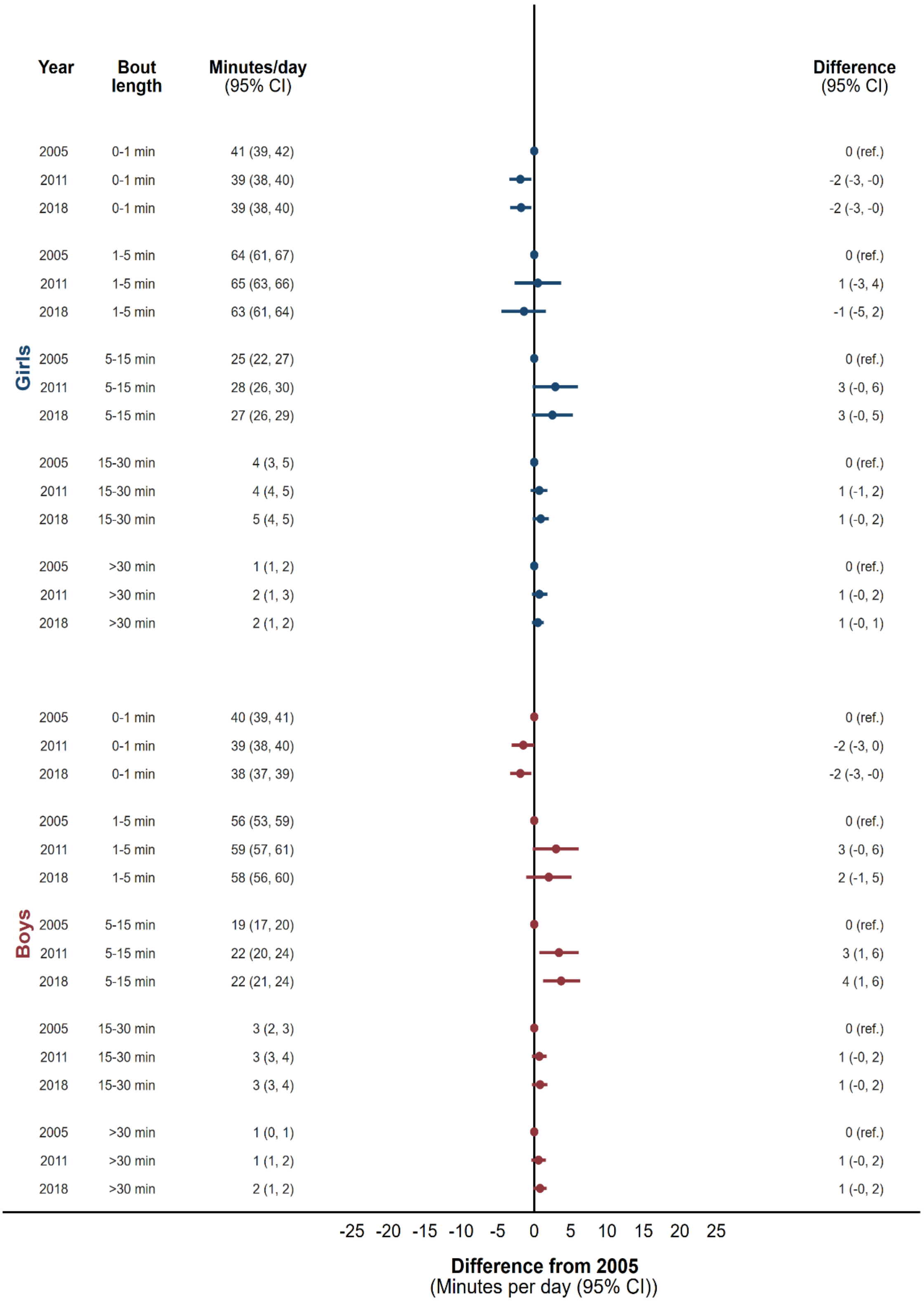

Girls; n = 1557, boys; n = 1589. Adjusted for age, minutes of daylight, and wear-time during school. Inclusion criteria shown in Supplemental Table 1.

Supplemental Figure 10. School sedentary time accumulation patterns in 15-year old boys and girls

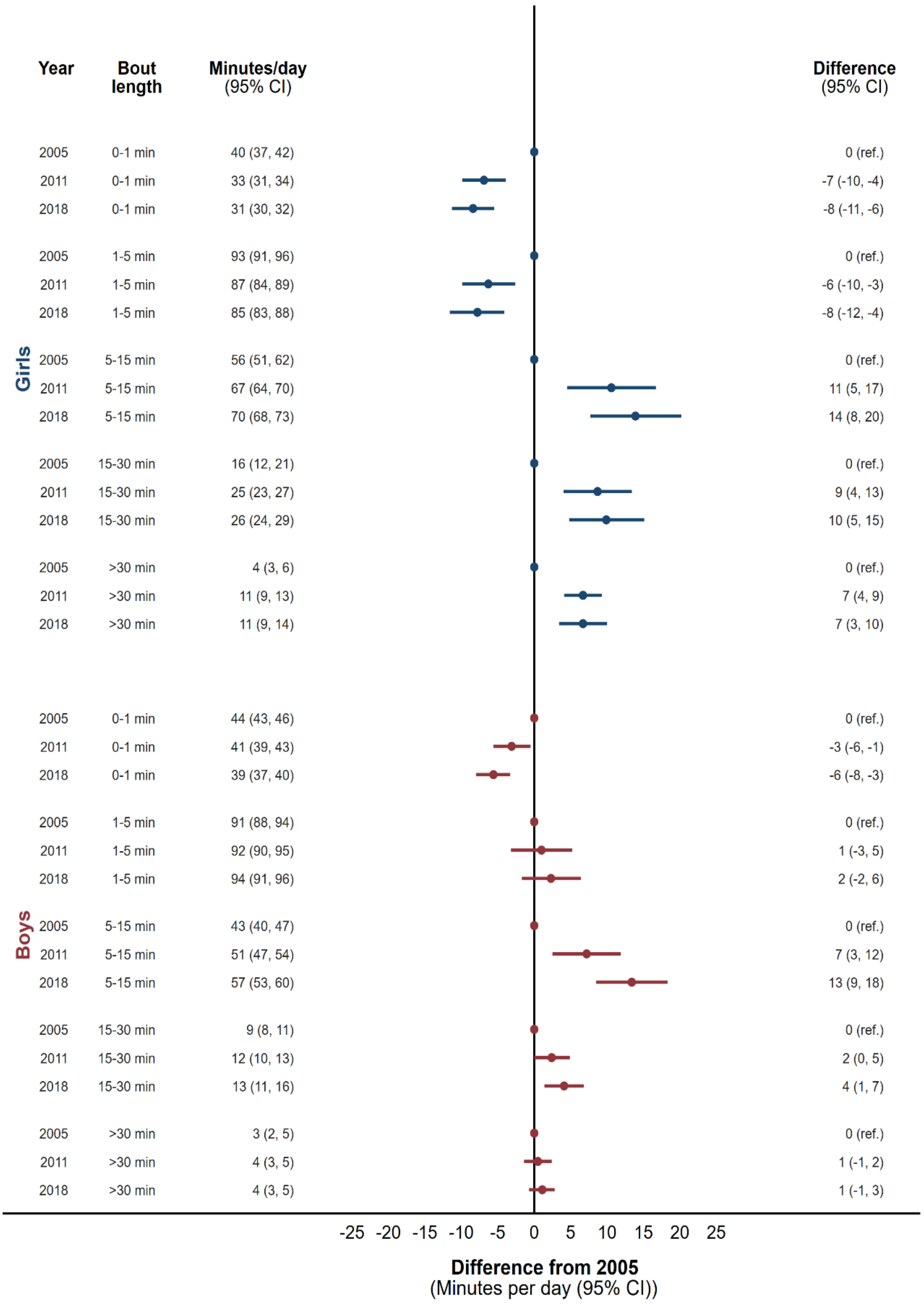

Girls; n = 1051, boys; n = 937. Adjusted for age, minutes of daylight, and wear-time during school. Inclusion criteria shown in Supplemental Table 1.

Supplemental Figure 11. Afterschool sedentary time accumulation patterns in 9-year old boys and girls

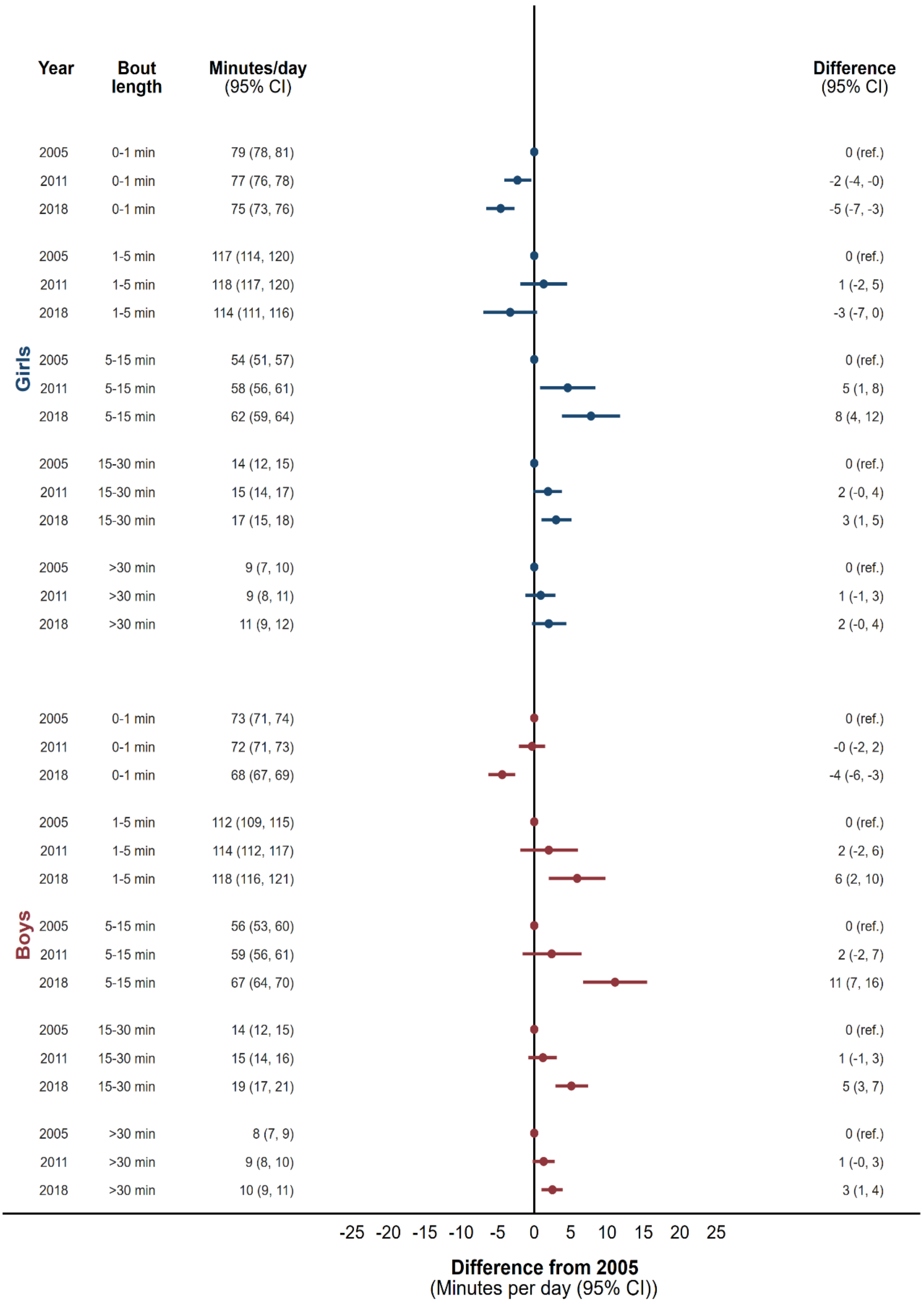

Girls; n = 1562, boys; n = 1594. Adjusted for age, minutes of daylight, and wear-time after school. Inclusion criteria shown in Supplemental Table 1.

Supplemental Figure 12. Afterschool sedentary time accumulation patterns in 15-year old boys and girls

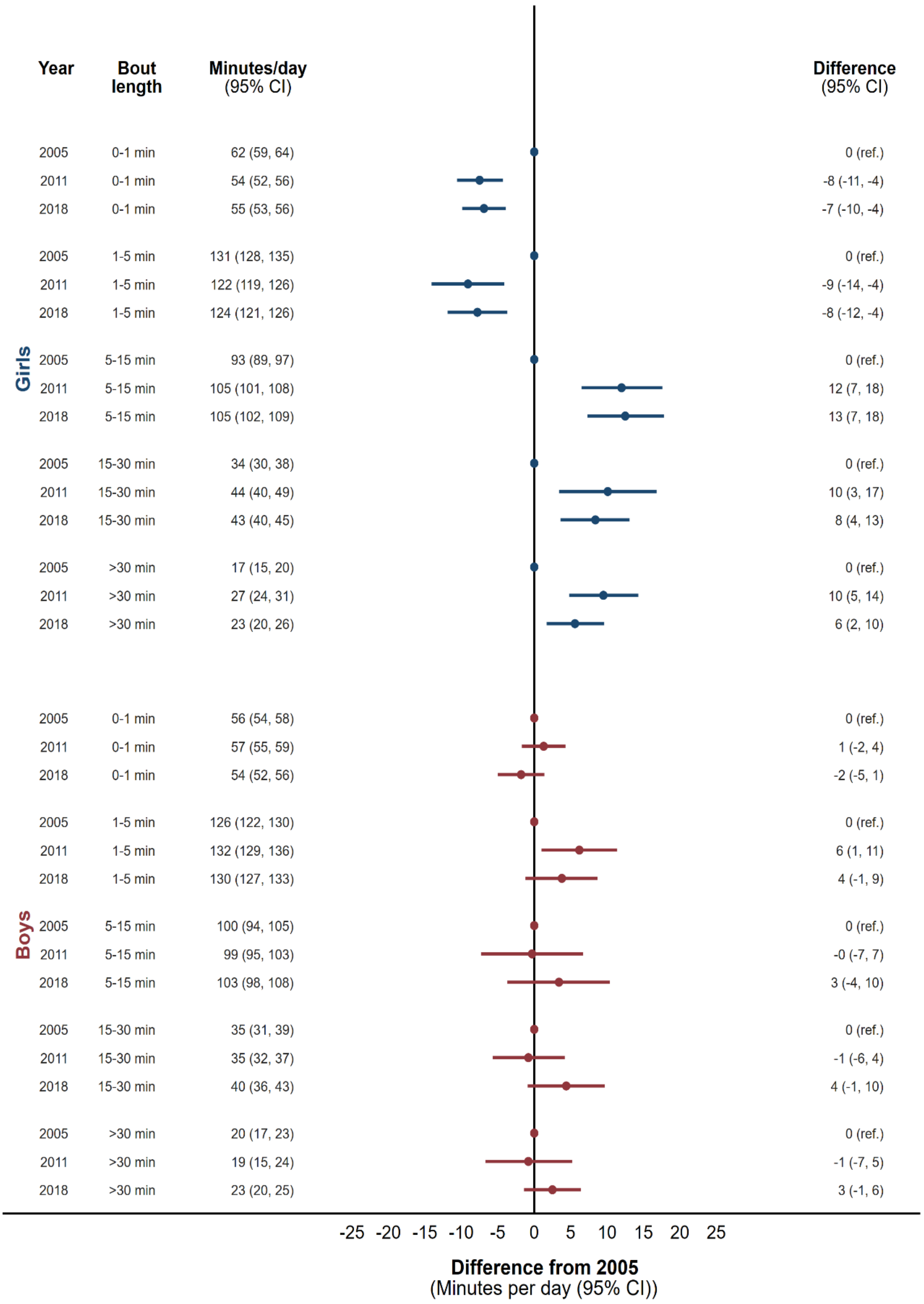

Girls; n = 1054, boys; n = 933. Adjusted for age, minutes of daylight, and wear-time after school. Inclusion criteria shown in Supplemental Table 1.

References

1. Grydeland M, Hansen BH, Ried-Larsen M, Kolle E, Anderssen SA. Comparison of three generations of ActiGraph activity monitors under free-living conditions: do they provide comparable assessments of overall physical activity in 9-year old children? *BMC Sports Sci Med Rehabil* 2014; **6**: 26.
